# How quantum brain biology can rescue conscious free will

**DOI:** 10.3389/fnint.2012.00093

**Published:** 2012-10-12

**Authors:** Stuart Hameroff

**Affiliations:** ^1^Department of Anesthesiology, Center for Consciousness Studies, University of ArizonaTucson, AZ, USA; ^2^Department of Psychology, Center for Consciousness Studies, University of ArizonaTucson, AZ, USA

**Keywords:** microtubules, free will, consciousness, Penrose-Hameroff Orch OR, volition, quantum computing, gap junctions, gamma synchrony

## Abstract

Conscious “free will” is problematic because (1) brain mechanisms causing consciousness are unknown, (2) measurable brain activity correlating with conscious perception apparently occurs too late for real-time conscious response, consciousness thus being considered “epiphenomenal illusion,” and (3) determinism, i.e., our actions and the world around us seem algorithmic and inevitable. The Penrose–Hameroff theory of “orchestrated objective reduction (Orch OR)” identifies discrete conscious moments with quantum computations in microtubules inside brain neurons, e.g., 40/s in concert with gamma synchrony EEG. Microtubules organize neuronal interiors and regulate synapses. In Orch OR, microtubule quantum computations occur in integration phases in dendrites and cell bodies of integrate-and-fire brain neurons connected and synchronized by gap junctions, allowing entanglement of microtubules among many neurons. Quantum computations in entangled microtubules terminate by Penrose “objective reduction (OR),” a proposal for quantum state reduction and conscious moments linked to fundamental spacetime geometry. Each OR reduction selects microtubule states which can trigger axonal firings, and control behavior. The quantum computations are “orchestrated” by synaptic inputs and memory (thus “Orch OR”). If correct, Orch OR can account for conscious causal agency, resolving problem 1. Regarding problem 2, Orch OR can cause temporal non-locality, sending quantum information backward in classical time, enabling conscious control of behavior. Three lines of evidence for brain backward time effects are presented. Regarding problem 3, Penrose OR (and Orch OR) invokes non-computable influences from information embedded in spacetime geometry, potentially avoiding algorithmic determinism. In summary, Orch OR can account for real-time conscious causal agency, avoiding the need for consciousness to be seen as epiphenomenal illusion. Orch OR can rescue conscious free will.

## Introduction: three problems with free will

We have the sense of conscious control of our voluntary behaviors, of free will, of our mental processes exerting causal actions in the physical world. But such control is difficult to scientifically explain for three reasons:

### Consciousness and causal agency

What is meant, exactly, by “we” (or “I”) exerting conscious control? The scientific basis for consciousness, and “self,” are unknown, and so a mechanism by which conscious agency may act in the brain to exert causal effects in the world is also unknown.

### Does consciousness come too late?

Brain electrical activity correlating with conscious perception of a stimulus apparently can occur *after* we respond to that stimulus, seemingly consciously. Accordingly, science and philosophy generally conclude that we act non-consciously, and have subsequent false memories of conscious action, and thus cast consciousness as epiphenomenal and illusory (e.g., Dennett, 1991; Wegner, 2002).

### Determinism

Even if consciousness and a mechanism by which it exerts real-time causal action came to be understood, those specific actions could be construed as entirely algorithmic and inevitably pre-ordained by our deterministic surroundings, genetics and previous experience.

We do know that causal behavioral action and other cognitive functions derive from brain neurons, and networks of brain neurons, which integrate inputs to thresholds for outputs as axonal firings, which then collectively control behavior. Such actions may be either (seemingly, at least) conscious/voluntary, or non-conscious (i.e., reflexive, involuntary, or “auto-pilot”). The distinction between conscious and non-conscious activity [the “neural correlate of consciousness (NCC)”] is unknown, but often viewed as higher order emergence in computational networks of integrate-and-fire neurons in cortex and other brain regions (Scott, 1995). Cortical-cortical, cortical-thalamic, brainstem and limbic networks of neurons connected by chemical synapses are generally seen as neurocomputational frameworks for conscious activity, (e.g., Baars, 1988; Crick and Koch, 1990; Edelman and Tononi, 2000; Dehaene and Naccache, 2001), with pre-frontal and pre-motor cortex considered to host executive functions, planning and decision making.

But even if specific networks, neurons, membrane, and synaptic activities involved in consciousness were completely known, questions would remain. Aside from seemingly occurring too late for conscious control, neurocomputational activity fails to: (1) distinguish between conscious and non-conscious (“auto-pilot”) cognition, (2) account for long-range gamma synchrony electro-encephalography (“EEG”), the best measurable NCC (Singer and Gray, 1995), for which gap junction electrical synapses are required, (3) account for “binding” of disparate activities into unified percepts, (4) consider scale-invariant (“fractal-like,” “1/f”) brain dynamics and structure, and (5) explain the “hard problem” of subjective experience (e.g., Chalmers, 1996). A modified type of neuronal network can resolve some of these issues, but to fully address consciousness and free will, something else is needed. Here I propose the missing ingredient is finer scale, deeper order, molecular-level quantum effects in cytoskeletal microtubules inside brain neurons.

In particular, the Penrose–Hameroff “Orch OR” model suggests that quantum computations in microtubules inside brain neurons process information and regulate membrane and synaptic activities. Microtubules are lattice polymers of subunit proteins called “tubulin.” Orch OR proposes tubulin states in microtubules act as interactive information “bits,” and also as quantum superpositions of multiple possible tubulin states (e.g., quantum bits or qubits). During integration phases, tubulin qubits interact by entanglement, evolve and compute by the Schrödinger equation, and then reduce, or collapse to definite states, e.g., after 25 ms in gamma synchrony. The quantum state reduction is due to an objective threshold [“objective reduction (OR)”] proposed by Penrose, accompanied by a moment of conscious awareness. Synaptic inputs and other factors “orchestrate” the microtubule quantum computations, hence “orchestrated objective reduction (Orch OR).”

Orch OR directly addresses conscious causal agency. Each reduction/conscious moment selects particular microtubule states which regulate neuronal firings, and thus control conscious behavior. Regarding consciousness occurring “too late,” quantum state reductions seem to involve temporal non-locality, able to refer quantum information both forward and backward in what we perceive as time, enabling real-time conscious causal action. Quantum brain biology and Orch OR can thus rescue free will.

## Consciousness, brain, and causality

Consciousness involves awareness, phenomenal experience (composed of what philosophers term “qualia”), sense of self, feelings, apparent choice and control of actions, memory, a model of the world, thought, language, and, e.g., when we close our eyes, or meditate, internally-generated images and geometric patterns. But what consciousness actually *is* remains unknown.

**Figure 1:**
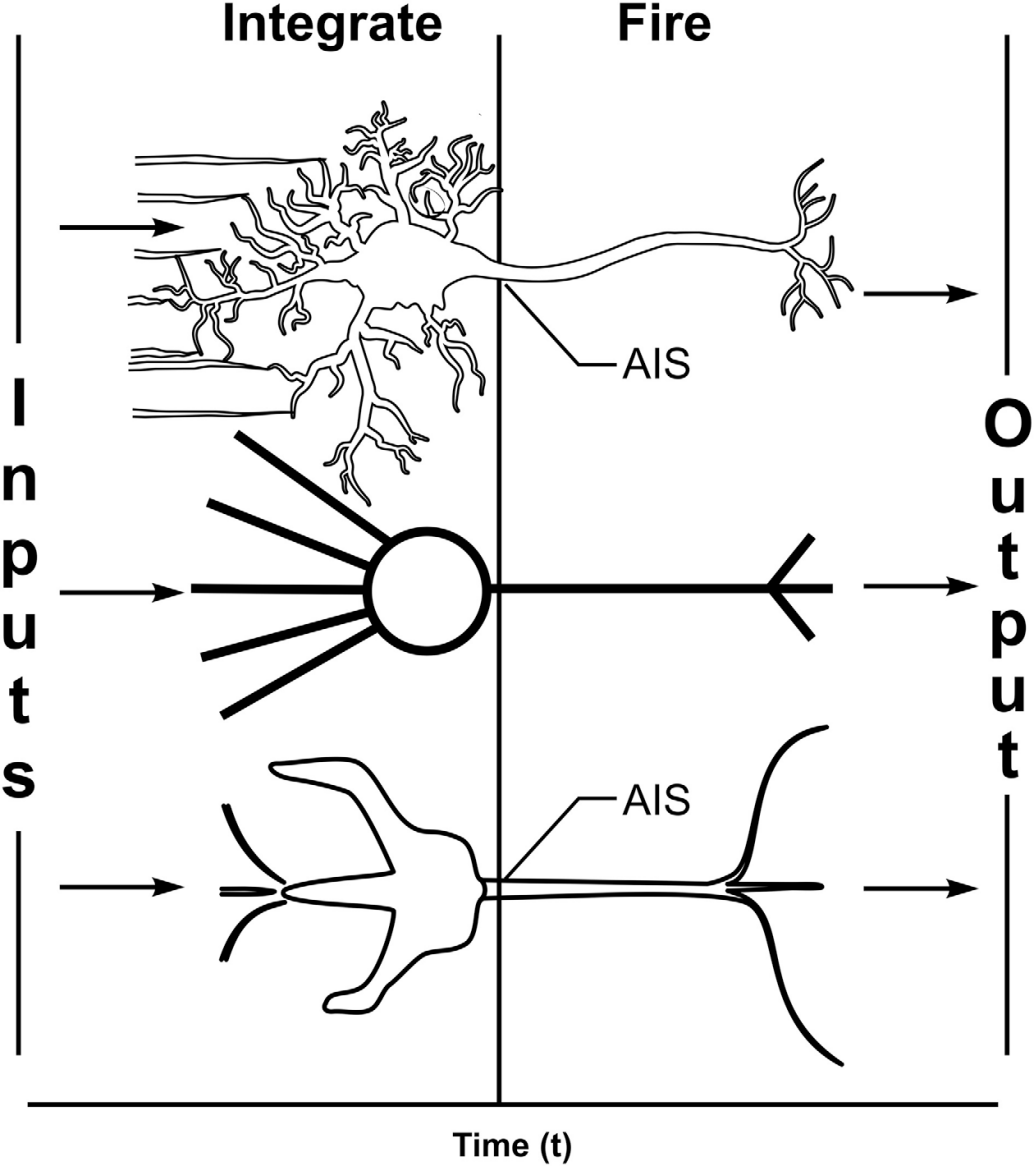
**Three characterizations of integrate-and-fire neurons. Top:** Biological neuron with multiple dendrites and one cell body (soma) receive and integrate synaptic inputs as membrane potentials which are compared to a threshold at the axon initiation segment (AIS). If threshold is met, axonal spikes/firings are triggered along a single axon which branches distally to convey outputs. **Middle:** computer-based artificial neuron (e.g., a “perceptron,” Rosenblatt, 1962) with multiple weighted inputs and single branched output. **Bottom:** model neuron (see subsequent figures) showing the same essential features with three inputs on one dendrite and single axonal output which branches distally.

Most scientists and philosophers view consciousness as an emergent property of complex computation among networks of the brain's 100 billion “integrate-and-fire” neurons. In digital computers, discrete voltage levels represent information units (e.g., “bits”) in silicon logic gates. McCulloch and Pitts (1943) arranged logic gates as integrate-and-fire silicon neurons, leading to “perceptrons” (Rosenblatt, 1962; Figure 1) and self-organizing “artificial neural networks” capable of learning and self-organized behavior. Similarly, according to the standard “Hodgkin and Huxley” (1952) model, biological neurons are “integrate-and-fire” threshold logic device in which multiple branched dendrites and a cell body (soma) receive and integrate synaptic inputs as membrane potentials. The integrated potential is then compared to a threshold potential at the axon hillock, or axon initiation segment (AIS). When AIS threshold is reached by the integrated potential, an all-or-none action potential “firing,” or “spike” is triggered as output, conveyed along the axon to the next synapse. Axonal firings can manifest will and behavior, e.g., causing other neurons to move muscles or speak words.

**Figure 2:**
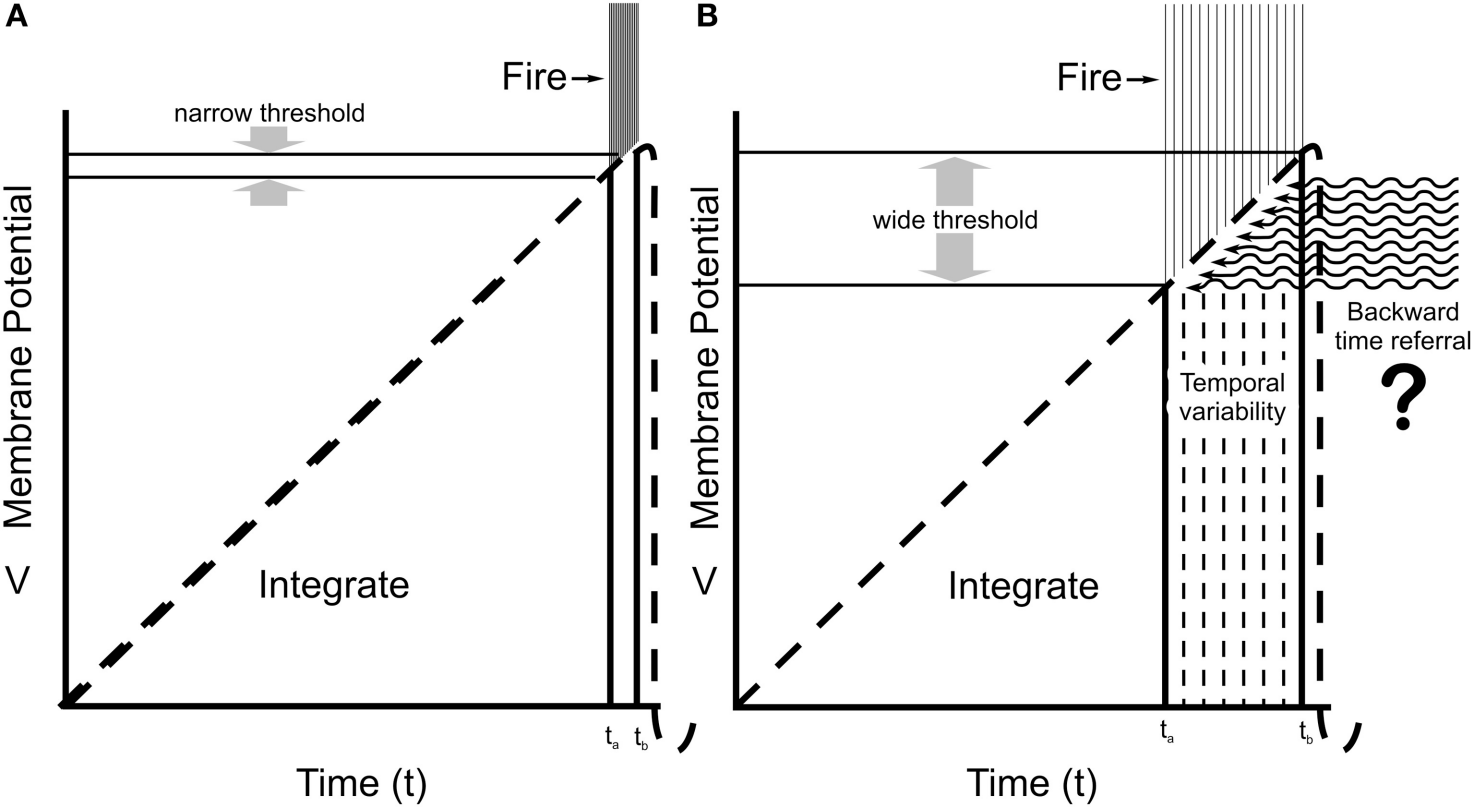
**Integrate-and-fire neuronal behaviors. (A)** The Hodgkin–Huxley model predicts integration by membrane potential in dendrites and soma reach a specific, narrow threshold potential at the proximal axon (AIS), and fire with very low temporal variability (small *t*_b_–*t*_a_) for given inputs. **(B)** Recordings from cortical neurons in awake animals (Naundorf et al., 2006) show a large variability in effective firing threshold and timing. Some unknown “x-factor” (related to consciousness?) exerts causal influence on firing and behavior. Here, quantum temporal non-locality results in backward time referral, suggested as the “x-factor” modulating firing threshold.

Some contend that consciousness emerges from axonal firing outputs, “volleys,” or “explosions” from complex neurocomputation (Koch, 2004; Malach, 2007). But coherent axonal firings are preceded and caused by synchronized dendritic/somatic integrations, suggesting consciousness involves neuronal dendrites and cell bodies/soma, i.e., in integration phases of “integrate-and-fire” sequences (Pribram, 1991; Eccles, 1992; Woolf and Hameroff, 2001; Tononi, 2004). Integration implies merging and consolidation of multiple input sources to one output, e.g., chemical synaptic inputs integrated toward threshold for firing, commonly approximated as linear summation of dendritic/somatic membrane potentials. However actual integration is active, not passive, and involves complex logic and signal processing in dendritic spines, branch points and local regions, amplification of distal inputs, and changing firing threshold at the AIS trigger zone (Shepherd, 1996; Sourdet and Debanne, 1999; Poirazi and Mel, 2001). Dendrites and soma are primary sources of EEG, and sites of anesthetic action which erase consciousness with little or no effects on axonal firing capabilities. Arguably, dendritic/somatic integration is closely related to consciousness, with axonal firings the outputs of conscious (or non-conscious) processes. Nonetheless, according to the Hodgkin–Huxley model, integration is assumed to be completely algorithmic and deterministic (Figure 2A), leaving no apparent room for conscious free will.

However, Naundorf et al. (2006) showed that firing threshold in cortical neurons in brains of awake animals (compared to neurons in slice preparations) varies widely on a spike-to-spike, firing-to-firing basis. Some factor other than the integrated AIS membrane potential contributes to firing, or not firing (Figure 2B). Firings control behavior. This “x-factor,” modulating integration and adjusting firing threshold and timing, is perfectly positioned for causal action, for conscious free will. What might it involve? Figure 2B indicates possible modification of integration and firing threshold by backward time referral.

**Figure 3:**
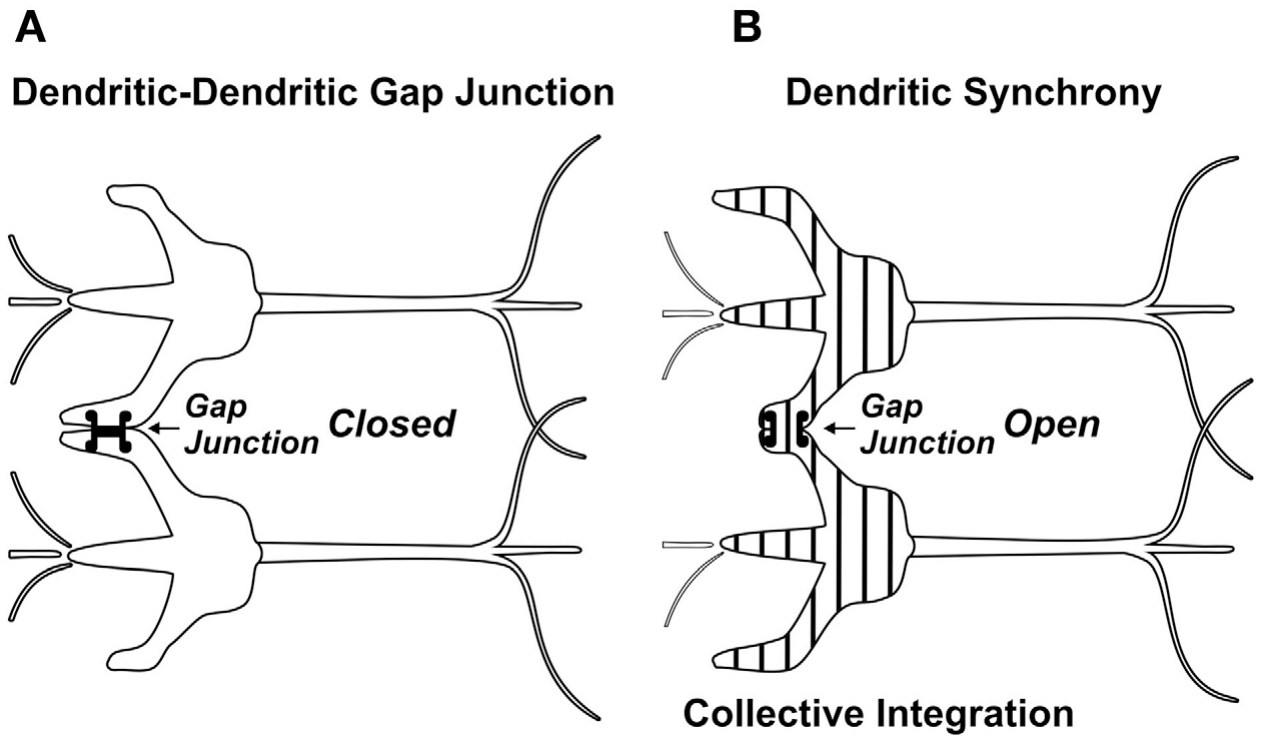
**(A)** Dendrites of adjacent neurons linked by gap junction which remain closed. The gap junction connection is “sideways,” lateral to the flow of synaptic information. **(B)** Dendritic-dendritic gap junction open, synchronizing (vertical stripes) electrophysiology and enabling collective integration among gap junction-connected neurons.

**Figure 4:**
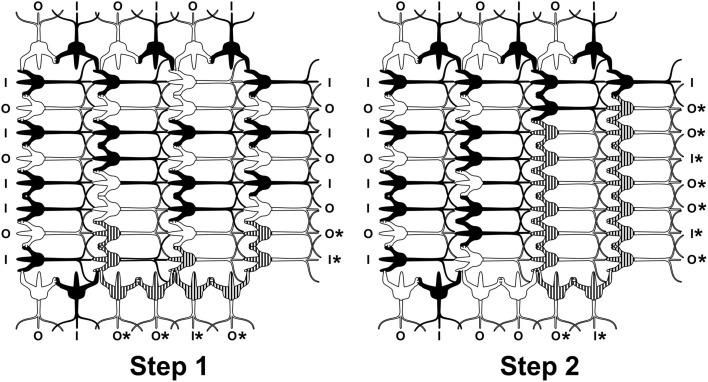
**Two timesteps in a neurocomputational network of integrate-and-fire neurons.** Inputs come from left, outputs go to top, bottom and right. Dendritic-dendritic gap junctions may open, e.g., between striped dendrites and soma to form “synchronized webs.” As gap junctions open and close, the synchronized web can move through the network, e.g., Step 1, 2. Mobile webs are candidates for the neural correlates of consciousness (NCC). Outputs marked by ^*^ reflect collective integration and suggest conscious causal agency.

Anatomically, a source for integration and firing threshold modification comes from lateral connections among neurons via gap junctions, or electrical synapses (Figure 3). Gap junctions are membrane protein complexes in adjacent neurons (or glia) which fuse the two cells and synchronize their membrane polarization states e.g., in gamma synchrony EEG (Dermietzel, 1998; Draguhn et al., 1998; Galarreta and Hestrin, 1999; Bennett and Zukin, 2004; Fukuda, 2007), the best measurable NCC (Gray and Singer, 1989; Fries et al., 2002; Kokarovtseva et al., 2009). Gap junction-connected cells also have continuous intracellular spaces, as open gap junctions between cells act like windows, or doors between adjacent rooms. Neurons connected by dendritic-dendritic gap junctions have synchronized local field potentials (EEG) in integration phase, but not necessarily synchronous axonal firing outputs. Thus gap junction synchronized dendritic networks can collectively integrate inputs, and provide an x-factor in selectively controlling firing outputs (Hameroff, 2010). Gap junction dynamics may also enable mobile agency in the brain. As gap junctions open and close, synchronized zones of collective integration and conscious causal agency can literally move through the brain, modulating integration, firing thresholds and behavior (Figure 4; Hameroff, 2010; Ebner and Hameroff, 2011). As consciousness can occur in different brain locations at different times, the NCC may be a mobile zone exerting conscious causal agency in various brain regions at different times.

But why would such causal agency be conscious? And with membranes synchronized, how do gap junction-connected neurons share and integrate information? Evidence points to the origins of behavior and consciousness at a deeper order, finer scale within neurons, e.g., in cytoskeletal structures such as microtubules which organize cell interiors.

## A finer scale?

Single cell organisms like *Paramecium* swim about, avoid obstacles and predators, find food and mates, and have sex, all without any synaptic connections. They utilize cytoskeletal structures such as microtubules (in protruding cilia and within their internal cytoplasm) for sensing and movement. The single cell slime mold *Physarum polycephalum* sends out numerous tendrils composed of bundles of microtubules, forming patterns which, seeking food, can solve problems and escape a maze (e.g., Adamatzky, 2012). Observing the purposeful behavior of single cell creatures, neuroscientist Charles Sherrington (1957) remarked: “of nerve there is no trace, but perhaps the cytoskeleton might serve.”

**Figure 5:**
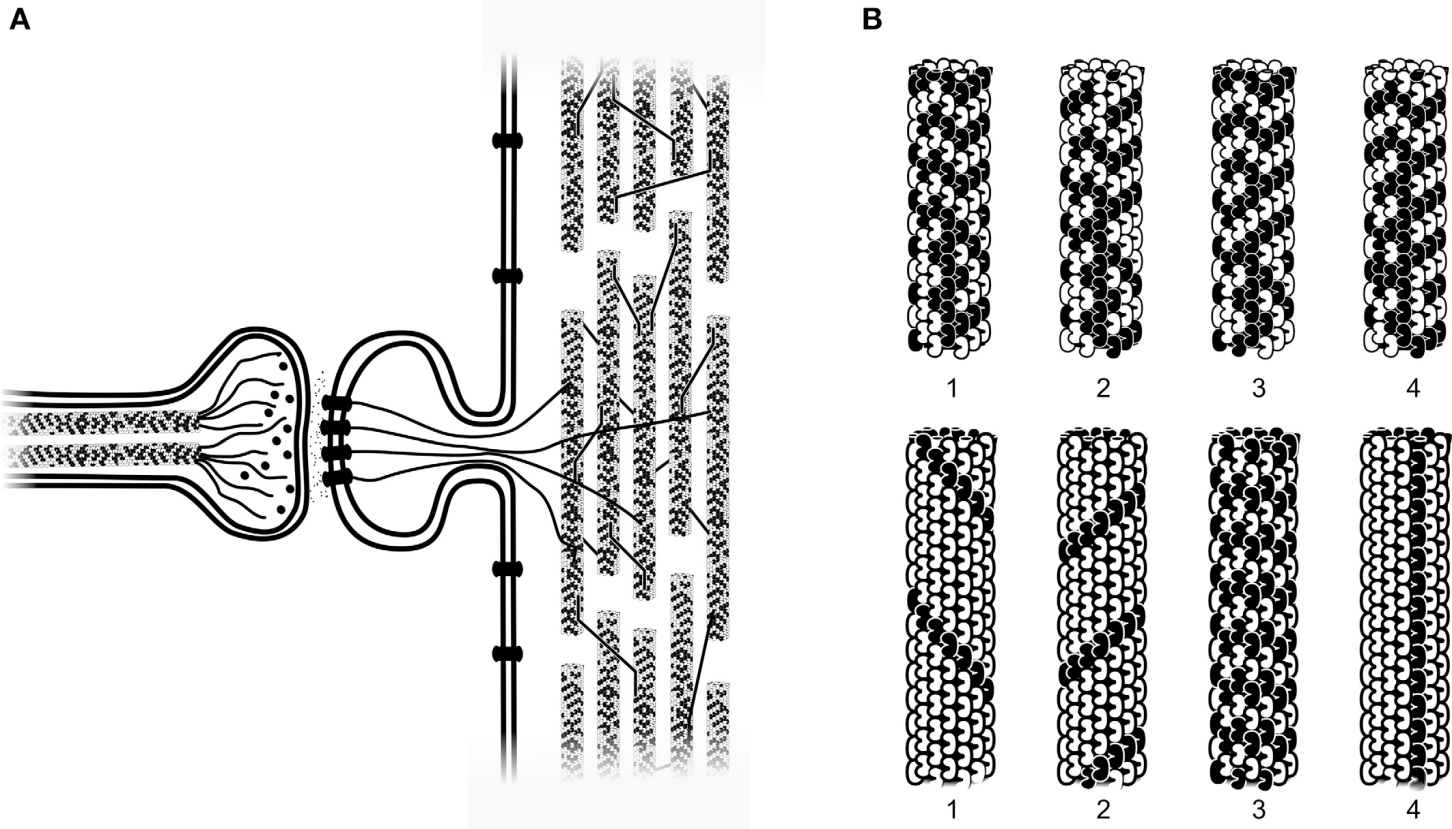
**(A)** Axon terminal (left) with two internal microtubules releasing neurotransmitters into synapse and onto receptors in membrane of dendritic spine. Actin filaments (as well as soluble second messengers, not shown) connect to cytoskeletal microtubules in main dendrite. Dendritic microtubules (right) are arranged in local networks, interconnected by microtubule-associated proteins (MAPs). **(B)** Larger scale showing two types of microtubule information processing. Top row: four timesteps in a microtubule automata simulation, each tubulin holding a bit state, switching e.g., at 10 megahertz (Rasmussen et al., 1990; Sahu et al., 2012). Bottom row: four topological bits in a microtubule. Information represented as specific helical pathways of conductance and information transfer. Microtubule mechanical resonances come into play (Hameroff et al., 2002; Sahu et al., 2012).

Interiors of animal cells are organized by the cytoskeleton, a scaffolding-like protein network of microtubules, microtubule-associated proteins (MAPs), actin and intermediate filaments (Figure 5A). Microtubules are cylindrical polymers 25 nm (nm = 10^−9^ m) in diameter, composed usually of 13 longitudinal protofilaments, each a chain of the peanut-shaped protein tubulin (Figure 5B). Microtubules self-assemble from tubulin, a ferroelectric dipole arranged within microtubules in two types of hexagonal lattices (A-lattice and B-lattice; Tuszynski et al., 1995), each slightly twisted, resulting in differing neighbor relationships among each subunit and its six nearest neighbors. Pathways along contiguous tubulins in the A-lattice form helical pathways which repeat every 3, 5, and 8 rows on any protofilament (the Fibonacci series; Figure 5B).

Each tubulin may differ from among its neighbors by genetic variability, post-translational modifications, binding of ligands and MAPs, and moment to moment dipole state transitions. Thus microtubules have enormous capacity for complex information representation and processing, are particularly prevalent in neurons (10^9^ tubulins/neuron), and uniquely stable and configured in dendrites and cell bodies (Craddock et al., 2012a). Microtubules in axons (and non-neuronal cells) are arrayed radially, extending continuously (all with the same polarity) from the centrosome near the nucleus, outward toward the cell membrane. However microtubules in dendrites and cell bodies are interrupted, of mixed polarity, stabilized, and arranged in local recursive networks suitable for learning and information processing (Figure 5A; Rasmussen et al., 1990).

Neuronal microtubules regulate synapses in several ways. They serve as tracks and guides for motor proteins (dynein and kinesin) which transport synaptic precursors from cell body to distal synapses, encountering, and choosing among several dendritic branch points and many microtubules. The guidance mechanism for such delivery, choosing the proper path, is unknown, but seems to involve the MAP tau as a traffic signal (placement of tau at specific sites on microtubules being the critical feature). In Alzheimer's disease, tau is hyperphosphorylated and dislodged from destabilized microtubules. Disruption of microtubules and formation of neurofibrillary tangles composed of free, hyperphosphorylated tau correlates with memory loss in Alzheimer's disease (Matsuyama and Jarvik, 1989; Craddock et al., 2012b), and post-anesthetic cognitive dysfunction (Craddock et al., 2012c).

Due to their lattice structure and direct involvement in organizing cellular functions, microtubules have been suggested to function as information processing devices. After Sherrington's (1957) broad observation about cytoskeletal information processing, Atema (1973) proposed that tubulin conformational changes propagate as signals along microtubules. Hameroff and Watt (1982) suggested that microtubule lattices act as two-dimensional Boolean computational switching matrices with input/output occurring via MAPs. Microtubule information processing has also been viewed in the context of cellular (“molecular”) automata in which tubulin states interact with hexagonal lattice neighbor tubulin states by dipole couplings, synchronized by biomolecular coherence as proposed by Fröhlich (1968, 1970, 1975); (Smith et al., 1984; Rasmussen et al., 1990). Simulations of microtubule automata based on tubulin states show rapid information integration and learning. Recent evidence indicates microtubules have resonances at frequency ranges from 10 kHz to 10 MHz, and possibly higher (Sahu et al., 2012). Topological computing can also occur in which helical pathways through the skewed hexagonal lattice are the relevant states, or bits (Figure 2B, bottom). Particular resonance frequencies may correlate with specific helical pathways.

With roughly 10^9^ tubulins per neuron switching at e.g., 10 MHz (10^7^), the potential capacity for microtubule-based information processing is 10^16^ operations/s *per neuron*. Integr-ation in microtubules (influenced by encoded memory), and synchronized in collective integration by gap junctions may be an x-factor in altering firing threshold and exerting causal agency in sets of synchronized neurons. But even a deeper order, finer scale microtubule-based process in a self-organizing zone of conscious agency would still be algorithmic and deterministic, and fail to address completely the problems of consciousness and free will.

And another problem looms.

## Is consciousness too late?

Several lines of evidence suggest that real time conscious action is an illusion, that we act non-consciously and have belated, false impressions of conscious causal action. This implies that free will does not exist, that consciousness is epiphenomenal, and that we are, as Huxley (1893/1986) bleakly summarized, “merely helpless spectators.” Apparent evidence against real-time conscious action includes the following:

### Sensory consciousness comes too late for conscious response

Neural correlates of conscious perception occur 150–500 ms after impingement on our sense organs, apparently too late for causal efficacy in seemingly conscious perceptions and willful actions, often initiated or completed within 100 ms after sensory impingement. Velmans (1991, 2000) listed a number of examples: analysis of sensory inputs and their emotional content, phonological, and semantic analysis of heard speech and preparation of one's own spoken words and sentences, learning and formation of memories, and choice, planning and execution of voluntary acts. Consequently, the subjective feeling of conscious control of these behaviors is deemed illusory (Dennett, 1991; Wegner, 2002).

In speech, evoked potentials (EPs) indicating conscious word recognition occur about 400 ms after auditory input, however semantic meaning is appreciated (and response initiated) after only 200 ms. As Velmans points out, only two phonemes are heard by 200 ms, and an average of 87 words share their first two phonemes. Even when contextual effects are considered, semantic processing and initiation of response occur before conscious recognition (Van Petten et al., 1999).

Gray (2004) observes that in tennis “The speed of the ball after a serve is so great, and the distance over which it has to travel so short, that the player who receives the serve must strike it back before he has had time consciously to see the ball leave the server's racket. Conscious awareness comes too late to affect his stroke.” McCrone (1999): “[for] tennis players … facing a fast serve … even if awareness were actually instant, it would still not be fast enough ….” Nonetheless tennis players claim to see the ball consciously before they attempt to return it.

### Readiness potentials

**Figure 6:**
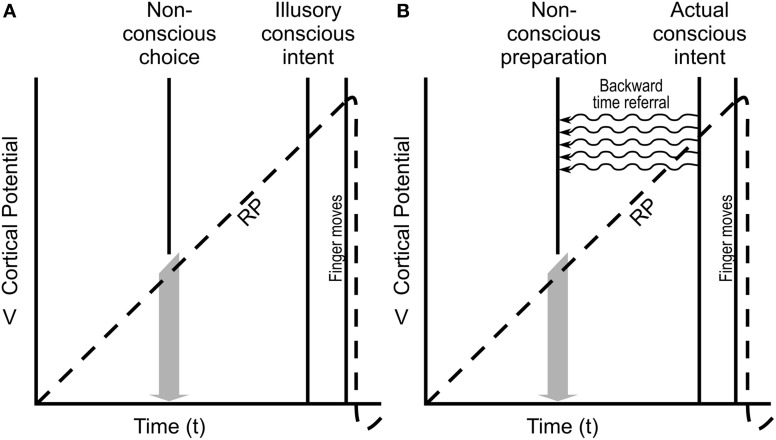
**The “readiness potential (RP)” (Libet et al., 1983). (A)** Cortical potentials recorded from a subject instructed to move his/her hand whenever he/she feels ready, and to note when the decision was made (Conscious intent), followed quickly by the finger actually moving. (Time between Conscious intent, and finger moving is fixed.) Readiness potential, RP, preceding Conscious intent is generally interpreted as representing the Non-conscious choice to move the finger, with Conscious intent being illusion. **(B)** Assuming RP is necessary preparation for conscious finger movement, Actual conscious intent could initiate the earlier RP by (quantum) temporal non-locality and backward time referral, enabling preparation while preserving real time conscious intent and control.

Kornhuber and Deecke (1965) recorded brain electrical activity over pre-motor cortex in subjects who were asked to move their finger randomly, at no prescribed time. They found that brain electrical activity preceded finger movement by ~800 ms, calling this activity the *readiness potential* (“RP,” Figure 6A). Libet and colleagues (1983) repeated the experiment, except they also asked subjects to note precisely when they consciously decided to move their finger. (To do so, and to avoid delays caused by verbal report, Libet et al. used a rapidly moving clock and asked subjects to note when on the clock they consciously decided to move their finger). This conscious decision came ~200 ms before actual finger movement, hundreds of milliseconds after onset of the RP. Libet and many authorities concluded that the RP represented non-conscious determination of movement, that many seemingly conscious actions are actually initiated by nonconscious processes, and that conscious intent was an illusion. Consciousness apparently comes too late. However, as shown in Figure 6B, temporal non-locality enabling backward time referral of (quantum) information from the moment of conscious intent can account for necessary RP preparation.

And yet we feel as though we act consciously in real time. To account for this paradox, Dennett (1991); (cf. Dennett and Kinsbourne, 1992) described real time conscious perception and action as retrospective construction, as illusion. His *multiple drafts* model proposed sensory inputs and cognitive processing produced tentative contents under continual revision, with the definitive, final edition only inserted into memory, overriding previous drafts (“Orwellian Revisionism” after George Orwell's fictional, retroactive “Ministry of Truth” in the novel 1984). Perceptions are edited and revised over hundreds of milliseconds, a final version inserted into memory. In this view (more or less the standard in modern philosophy and neuroscience) the brain retrospectively creates content or judgment, e.g., of real time conscious control which is recorded in memory as veridical truth. In other words, we act non-consciously in real time, but then falsely remember acting consciously. Consciousness, in this view, is an epiphenomenal illusion occurring after-the-fact. We are living in the past.

**Figure 7:**
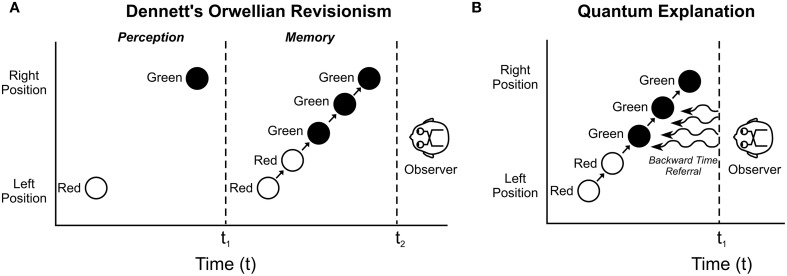
**In the “color phi” phenomenon (Kolers and von Grunau, 1976).** A red circle appears on the left side of a screen, disappears, and then, a fraction of a second later, a green circle appears on the right side. An observer consciously “sees” a red circle moving continuously from left to right, changing to green halfway across. **(A)** According to Dennett's “Orwellian Revisionism,” the brain constructs, or fills in the movement and transition after the fact, and inserts a constructed perception into memory. Real-time perception is not conscious. **(B)** In a “Quantum Explanation,” temporal non-locality and backward time referral allow real-time, veridical conscious perception.

For example in the “color phi” effect (Kolers and von Grunau, 1976) a red spot appears briefly on the left side of a screen, followed after a pause by a green spot on the right side. Conscious observers report one spot moving back and forth, changing to green halfway across the screen, the brain seemingly “filling in” (Figure 7). Yet after a sequence of such observations, if the spot on the right is suddenly red (instead of green), the subject is not fooled and fills in continuously with red halfway across. Does the brain *know in advance* to which color the dot will change? No, says Dennett. The brain fills in the proper color in a subsequent draft, and belatedly imprints it into conscious memory. Consciousness occurs after the fact (Figure 7A). Any conscious response to the color change would occur well after presentation, dooming free will. However a quantum explanation with temporal non-locality and backward time referral enables constructive “filling in” from near future brain activity, allowing real time conscious perception (Figure 7B). Is there any evidence for backward time effects in the brain?

## Backward time effects in the brain? three lines of evidence

### Libet's “open brain” sensory experiments

**Figure 8:**
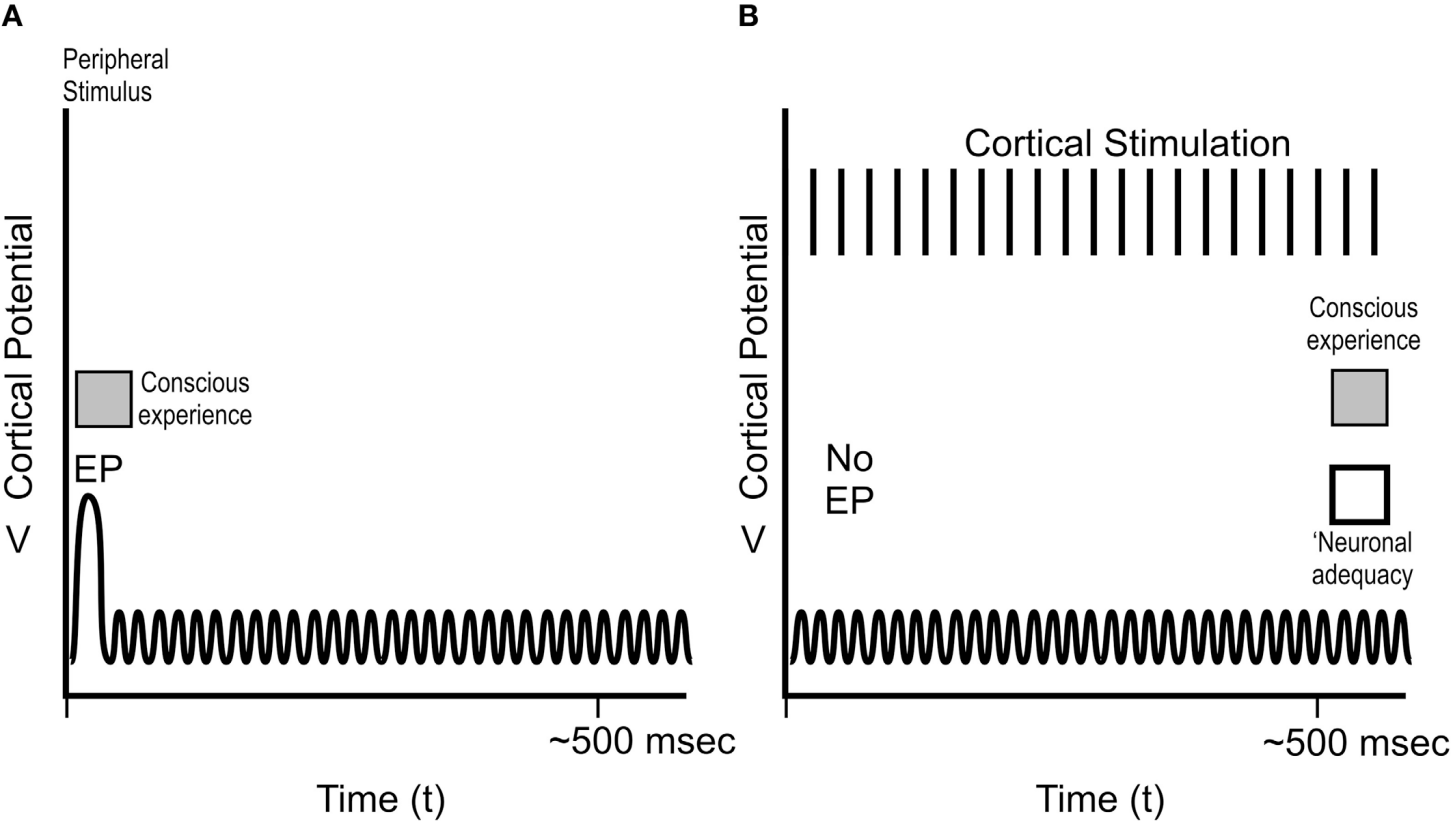
**Cortical potentials in Libet's sensory experiments. (A)** Peripheral stimulation, e.g., at the hand, results in near-immediate conscious experience of the stimulation, an evoked potential EP at ~30 ms in the “hand area” of somatosensory cortex, and several 100 ms of ongoing cortical electrical activity. **(B)** Direct cortical activity of the somatosensory cortical hand area for several 100 ms results in no EP, ongoing cortical activity, and conscious sensory experience of the hand, but only after ~500 ms. Libet termed the 500 ms of cortical activity resulting in conscious experience “neuronal adequacy.”

**Figure 9:**
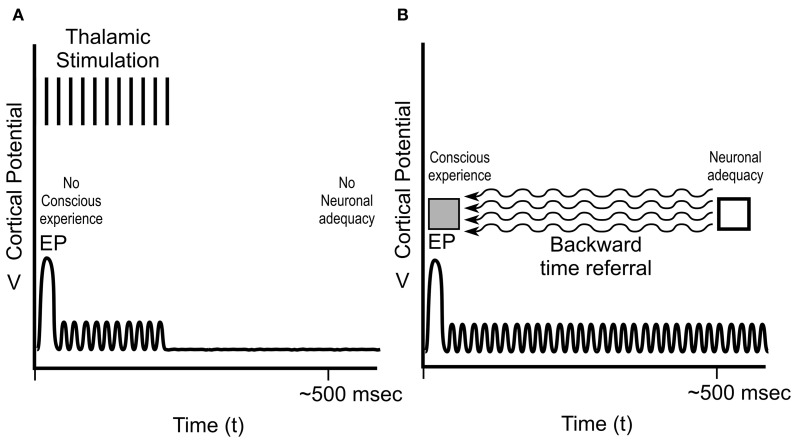
**Libet's sensory experiments, continued. (A)** Libet et al. stimulated medial lemniscus of thalamus in the sensory pathway to produce an EP (~30 ms) in somatosensory cortex, but only brief post-EP stimulation, resulting in only brief cortical activity. There was no apparent “neuronal adequacy,” and no conscious experience. An EP and several 100 ms of post-EP cortical activity (neuronal adequacy) were required for conscious experience at the time of EP. **(B)** To account for his findings, Libet concluded that subjective information was referred backward in time from neuronal adequacy (~500 ms) to the EP.

In addition to volitional studies (moving a finger), Libet and colleagues studied the timing of conscious sensory experience in awake, cooperative patients undergoing brain surgery with local anesthesia (e.g., Libet et al., 1964, 1979; Libet, 2004). With his neurosurgical colleagues, in these patients Libet was able to record from, and stimulate specific areas of somatosensory cortex, e.g., corresponding to the skin of each patient's hand, and the hand itself (Figures 8 and 9), as well as communicate with the conscious patients.

As depicted in Figure 8A, peripheral stimulus, e.g., of the skin of the hand, resulted in an “EP” spike in the somatosensory cortical area for the hand ~30 ms after skin contact, consistent with the time required for a neuronal signal to travel from hand to spinal cord, thalamus, and brain. The stimulus also caused several 100 ms of ongoing cortical activity following the EP. Subjects reported conscious experience of the stimulus (using Libet's rapidly moving clock) near-immediately, e.g., at the time of the EP at 30 ms.

Libet also stimulated the “hand area” of subjects' brain somatosensory cortex directly (Figure 8B). This type of stimulation did *not* cause an EP spike, but did result in ongoing brain electrical activity. Conscious sensation referred to (“felt in”) the hand occurred, but only after stimulation and ongoing brain activity lasting up to 500 ms (Figure 8B). This requirement of ongoing, prolonged electrical activity (what Libet termed “neuronal adequacy”) to produce conscious experience (“Libet's 500 ms”) was subsequently confirmed by Amassian et al. (1991), Ray et al. (1999), Pollen (2004) and others.

But if hundreds of milliseconds of brain activity are required for neuronal adequacy, how can conscious sensory experience occur at 30 ms? To address this issue, Libet also performed experiments in which stimulation of thalamus resulted in an EP at 30 ms, but only brief ongoing activity, i.e., without neuronal adequacy (Figure 9A). No conscious experience occurred. Libet concluded that for real-time conscious perception (e.g., at the 30 ms EP), two factors were necessary: an EP at 30 ms, and several 100 ms of ongoing cortical activity (neuronal adequacy) *after* the EP. Somehow, apparently, the brain seems to know what will happen *after* the EP. Libet concluded the hundreds of milliseconds of ongoing cortical activity (“neuronal adequacy”) is the *sine qua non* for conscious experience—the NCC, even if it occurs after the conscious experience. To account for his results, he further concluded that subjective information is referred *backwards in time* from the time of neuronal adequacy to the time of the EP (Figure 9B). Libet's backward time assertion was disbelieved and ridiculed (e.g., Churchland, 1981; Pockett, 2002), but never refuted (Libet, 2002, 2003).

### Pre-sentiment and pre-cognition

Electrodermal activity measures skin impedance, usually with a probe wrapped around a finger, as an index of autonomic, sympathetic neuronal activity causing changes in blood flow and sweating, in turn triggered by emotional response in the brain. Over many years, researchers (Bierman and Radin, 1997; Bierman and Scholte, 2002; Radin, 2004) have published a number of well-controlled studies using electrodermal activity, fMRI and other methods to look for emotional responses, e.g., to viewing images presented at random times on a computer screen. They found, not surprisingly, that highly emotional (e.g., violent, sexual) images elicited greater responses than neutral, non-emotional images. But surprisingly, the changes occurred half a second to two seconds *before* the images appeared. They termed the effect pre-sentiment because the subjects were not consciously aware of the emotional feelings. Non-conscious emotional sentiment (i.e., feelings) appeared to be referred backward in time. These studies were published in the parapsychology literature, as mainstream academic journals refused to consider them.

Bem (2012) published “Feeling the future: experimental evidence for anomalous retroactive influences on cognition and affect” in the mainstream *J. Pers. Soc. Psychol*. The article reported on eight studies showing statistically significant backward time effects, most involving non-conscious influence of future emotional effects (e.g., erotic or threatening stimuli) on cognitive choices. Studies by others have reported both replication, and failure to replicate, the controversial results.

### Quantum delayed choice experiments

In the famous “double slit experiment,” quantum entities (e.g., photons, electrons) can behave as either waves, or particles, depending on the method chosen to measure them. Wheeler (1978) described a thought experiment in which the measurement choice (by a conscious human observer) was delayed until after the electron or other quantum entity passed though the slits, presumably as either wave or particle. Wheeler suggested the observer's delayed choice could retroactively influence the behavior of the electrons, e.g., as waves or particles. The experiment was eventually performed (Kim et al., 2000) and confirmed Wheeler's prediction; conscious choices can affect previous events, as long as the events had not been consciously observed in the interim.

In “delayed choice entanglement swapping,” originally a thought experiment proposed by Asher Peres (2000); Ma et al. (2012) went a step further. Entanglement is a characteristic feature of quantum mechanics in which unified quantum particles are separated but remain somehow connected, even over distance. Measurement or perturbation of one separated-but-still-entangled particle instantaneously affects the other, what Einstein referred to (mockingly) as “spooky action at a distance.” Despite its bizarre nature, entanglement has been demonstrated repeatedly, and is the foundation for quantum cryptography, quantum teleportation and quantum computing (Deutsch, 1985). In entanglement swapping, two pairs of unified/entangled particles are separated, and one from each pair is sent to two measurement devices, each associated with a conscious observer (“Alice” and “Bob,” as is the convention in such quantum experiments). The other entangled particle from each pair is sent to a third observer, “Victor.” How Victor decides to measure the two particles (as an entangled pair, or as separable particles) determines whether Alice and Bob observe them as entangled (showing quantum correlations) or separable (showing classical correlations). This happens even if Victor decides *after* Alice's and Bob's devices have measured them (but before Alice and Bob consciously view the results). Thus, conscious choice affects behavior of previously measured, but unobserved, events.

How can backward time effects be explained scientifically? The problem may be related to our perception of time in classical (non-quantum) physics. Anton Zeilinger, senior author on the Ma et al. study, said: “Within a naïve classical worldview, quantum mechanics can even mimic an influence of future actions on past events.”

## Time and conscious moments

What is time? St. Augustine remarked that when no one asked him, he knew what time was; however when someone asked him, he did not. The (“naïve”) worldview according to classical Newtonian physics is that time is either a process which flows, or a dimension in 4-dimensional space-time along which processes occur. But if time flows, it would do so in some medium or dimension (e.g., minutes per what?). If time is a dimension, why would processes occur unidirectionally in time? Yet we consciously perceive a unidirectional time-like reality. An alternative explanation is that time does not exist as process or dimension, but as a collage of discrete configurations of the universe, connected in some way by consciousness and memory (Barbour, 1999). This follows Leibniz “monads” (e.g., Rescher, 1991; c.f. Spinoza, 1677), momentary, snapshot-like arrangements of spatiotemporal reality based on Mach's principle that the universe has an underlying structure related to mass distribution (also a foundation of Einstein's general relativity). Whitehead (1929, 1933) expounded on Leibniz monads, conferring mental aspects to occasions occurring in a wider field of “proto-conscious experience” (“occasions of experience”). These views from philosophy and physics link consciousness to discrete events in the fine structure of physical reality.

Consciousness has also been seen as discrete events in psychology, e.g., James, (1890) “specious present, the short duration of which we are immediately and incessantly sensible” (though James was vague about duration, and also described a continual “stream of consciousness”). The “perceptual moment” theory of Stroud (1956) described consciousness as a series of discrete events, like sequential frames of a movie [modern film and video present 24–72 frames/s, 24–72 cycles/s, i.e., Hertz (“Hz”)]. Periodicities for perception and reaction times are in the range of 20–50 ms, i.e., gamma synchrony EEG (30–90 Hz). Slower periods, e.g., 4–7 Hz theta frequency, with nested gamma waves may correspond with saccades and visual gestalts (Woolf and Hameroff, 2001; VanRullen and Koch, 2003).

Support for consciousness as sequences of discrete events is also found in Buddhism, trained meditators describing distinct “flickerings” in their experience of pure undifferentiated awareness (Tart, 1995, pers. communication). Buddhist texts portray consciousness as “momentary collections of mental phenomena,” and as “distinct, unconnected and impermanent moments which perish as soon as they arise.” Buddhist writings even quantify the frequency of conscious moments. For example the Sarvaastivaadins (von Rospatt, 1995) described 6,480,000 “moments” in 24 h (an average of one “moment” per 13.3 ms, 75 Hz), and some Chinese Buddhism as one “thought” per 20 ms (50 Hz), both in gamma synchrony range.

Long-range gamma synchrony in the brain is the best measurable NCC. In surgical patients undergoing general anesthesia, gamma synchrony between frontal and posterior cortex is the specific marker which disappears with loss of consciousness and returns upon awakening ( Hameroff, 2006). In what may be considered enhanced or optimized levels of consciousness, high frequency (more than 80 Hz) phase coherent gamma synchrony was found spanning cortical regions in meditating Tibetan monks, at the highest amplitude ever recorded (Lutz et al., 2004). Faster rates of conscious moments may correlate with subjective perception of slower time flow, e.g., as in a car accident, or altered state. But what are conscious moments? Shimony (1993) recognized that Whitehead's occasions were compatible with quantum state reductions, or “collapses of the wave function.” Several lines of evidence suggest consciousness could be identified with sequences of quantum state reductions. What exactly are quantum state reductions?

## Consciousness and quantum state reduction

Reality is described by quantum physical laws which appear to reduce to classical rules (e.g., Newton's laws of motion) at certain scale limits, though those limits are unknown. According to quantum physical laws:

- Objects/particles may exist in two or more places or states simultaneously—more like waves than particles and governed by a quantum wavefunction. This property of multiple coexisting possibilities is known as quantum superposition.
- Multiple objects/particles can be unified, acting as a single coherent object governed by one wavefunction. If a component is perturbed, others *feel it* and react, e.g., in Bose-Einstein condensation.
- If unified objects are spatially separated they remain unified. This non-locality is also known as *quantum entanglement*.

But we don't see quantum superpositions in our macroscale world. How and why do quantum laws reduce to classical behavior? Various interpretations of quantum mechanics address this issue:

- *Copenhagen and the conscious observer*: In the early days of quantum mechanics, Bohr (1934/1987) and colleagues recognized that quantum superpositions persist until measured by a device (the “Copenhagen interpretation”, after Bohr's Danish origin). Wigner (1961) and von Neumann (1932/1955) further stipulated that the superposition continues in the device until the results are observed by a conscious human, that conscious observation “collapses the wave function.” These interpretations enabled quantum experiments to flourish, but put consciousness outside science, and failed to account for fundamental reality. Schrödinger (1935) took exception, posing his famous (“Schrödinger's cat”) thought experiment in which the fate of a cat in a box is tied to a quantum superposition, reasoning that, according to the Wigner and von Neumann interpretation, the cat would remain both dead and alive until the box is opened and observed by a conscious human. Despite the absurdity, limitations on quantum superposition remain unknown.
- *The multiple worlds* view suggests each superposition is a separation in reality, evolving to a new universe (Everett, 1957). There is no collapse, but an infinity of realities (and conscious minds) is required.
- David Bohm's interpretation (Bohm and Hiley, 1993) avoids reduction/collapse by postulating another layer of reality. Matter exists as objects guided by complex “pilot” waves of possibility.
- Henry Stapp (1993) views the universe as a single quantum wave function. Reduction within the brain is a conscious moment (akin to Whitehead's “occasion of experience”—Whitehead, 1929, 1933). Reduction/collapse *is* consciousness, but its cause and distinction between universal wave function and that within the brain are unclear.
- In decoherence theory (e.g., Zurek, 2003) any interaction (loss of isolation) of a quantum superposition with a classical system (e.g., through heat, direct interaction or information exchange) erodes the quantum system. But (1) the fate of isolated superpositions is not addressed, (2) no quantum system is ever truly isolated, (3) decoherence doesn't actually disrupt superposition, just buries it in noise, and (4) some quantum processes are *enhanced* by heat and/or noise.
- An objective threshold for quantum state reduction (*OR*) exists due to e.g., the number of superpositioned particles (GRW theory—Ghirardi et al., 1986) or a factor related to quantum gravity or underlying properties of spacetime geometry, as in the OR proposals of Károlyházy et al. (1986); Diȼsi (1989) and Penrose (1989, 1996). Penrose OR also includes consciousness, each OR event being associated with a moment of conscious experience.

**Figure 10:**
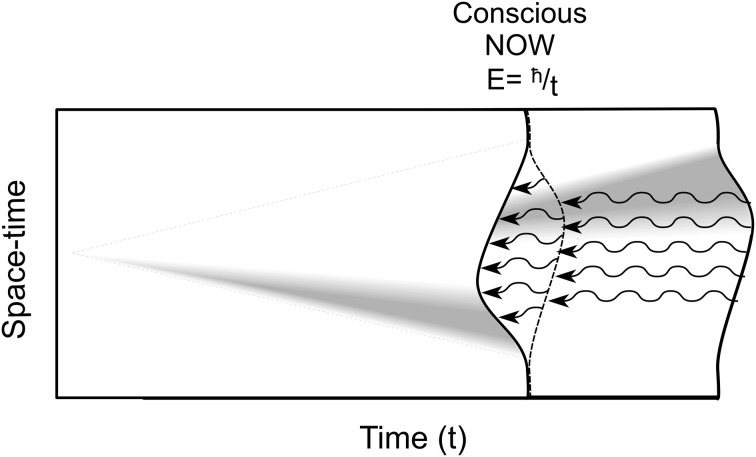
**Location or state of a particle/object is equivalent to curvature in underlying spacetime geometry.** From left, a superposition develops over time, e.g., a particle separating from itself, shown as simultaneous curvatures in opposite directions. The magnitude of the separation is related to *E*, the gravitational self-energy. At a particular time *t*, *E* reaches threshold by *E* = *ħ*/*t*, and spontaneous OR occurs, one particular curvature is selected. This OR event is accompanied by a moment of conscious experience (“NOW”), its intensity proportional to *E*. Each OR event also results in temporal non-locality, referring quantum information backward in classical time (curved arrows).

Penrose (1989, 1994) uniquely brings consciousness into physics, and directly approaches superpositioned objects as actual separations in underlying reality at its most basic level (fundamental space-time geometry at the Planck scale of 10^−33^ cm). Separation is akin to the multiple worlds view in which each possibility branches to form and evolve its own universe. However according to Penrose the space-time separations are unstable and (instead of branching off) spontaneously reduce (self-collapse) to one particular space-time geometry or another. This OR self-collapse occurs at a threshold given by *E* = *ħ*/*t*, where *E* is the magnitude (gravitational self-energy) of the superposition, e.g., the number of tubulins (*E* is also proportional to intensity of conscious experience), *ħ* is Planck's constant (over 2π), and *t* the time interval at which superposition E will self-reduce by OR, choosing classical states in a moment of consciousness (Figure 10).

Penrose *E* = *ħ*/*t* is related to the Heisenberg “uncertainty principle” which asserts a fundamental limit to the precision with which values for certain pairs of physical properties can be simultaneously known. The most common examples are uncertainty in position (*x*) and momentum (*p*) of a particle, given by their standard deviations (σ_*x*_ and σ_*p*_) whose product σ_*x*_σ_*p*_ is the uncertainty which must meet or exceed a fundamental limit related to *ħ*, Planck's constant over 2π. The uncertainty principle is thus usually written as σ_*x*_σ_*p*_ ≥ *ħ*/2. Uncertainty can pertain to properties other than position and momentum, and Penrose equated superposition/separation to uncertainty in the underlying structure of space-time itself. Heisenberg's uncertainty principle imposes a limit, causing quantum state reduction.

Space-time uncertainty is expressed as the gravitational self-energy *E*, the energy required for an object of mass *m* and radius *r* (or it's equivalent spacetime geometry) to separate *from itself* by a distance *a*. For Orch OR, *E* was calculated for superposition/separation of tubulin proteins at three levels, with three sets of *m, r, and a*. E was calculated for separation at the level of (1) the entire tubulin protein, (2) atomic nuclei within tubulin, and (3) nucleons (protons and neutrons) within tubulin atomic nuclei. Separation at the level of atomic nuclei (femtometers) was found to dominate, and used to calculate *E* (in terms of number of tubulins) for various values of time *t* corresponding with neurophysiology, e.g., 25 ms for gamma synchrony at 40 Hz. For a conscious event occurring at 25 ms, superposition/separation of 2 × 10^10^ tubulins are required, involving microtubules in roughly tens of thousands of neurons (Hameroff and Penrose, 1996a).

Particular states are chosen in OR due to (1) algorithmic quantum computing by the Schrödinger equation evolving toward *E* = *ħ*/*t*, and (2) influence in the OR process at the moment of *E* = *ħ*/*t*. According to Penrose, this influence, unlike randomness associated with measurement and decoherence, reflects “non-computable values” intrinsic to spacetime geometry. Thus conscious choices in OR (and Orch OR) are neither random nor algorithmically deterministic.

Quantum state reductions are essential to quantum computing which involves superposition of information states, e.g., both 1 *and* 0 (quantum bits, or “qubits”). Superpositioned qubits entangle and compute (by the Schrödinger equation) until reduction/collapse of each qubit to classical values (“bits”) occurs as the solution. In technological quantum computers, reduction occurs by measurement/observation, introducing a component of randomness. Superposition, entanglement and reduction are also essential to quantum cryptography and quantum teleportation technologies (Bennett and Wiesner, 1992; Bouwmeester et al., 1997; Macikic et al., 2002). Entanglement implies non-locality, e.g., that complementary quantum particles (electrons in coupled spin-up and spin-down pairs) remain somehow connected when spatially (or temporally) separated, each pair member reacting instantaneously to perturbation of its separated partner. Einstein initially objected to entanglement, as it would appear to require signaling faster than light, and thus violate special relativity. He famously termed it “spooky action at a distance,” and described a thought experiment (“Einstein, Podolsky, and Rosen (EPR)”; Einstein et al., 1935) in which each member of an entangled pair of superpositioned electrons (“EPR pairs”) would be sent in different directions, each remaining in superposition and entangled. When one electron was measured at its destination and, say, spin-up was observed, its entangled twin miles away would, according to the prediction, correspondingly reduce instantaneously to spin-down when measured. The issue was unresolved at the time of Einstein's death, but since the early 1980s (Aspect et al., 1982; Tittel et al., 1998) this type of experiment has been repeatedly confirmed through wires, fiber optic cables and via microwave beams through atmosphere. Strange as it seems, EPR entanglement is a fundamental feature of quantum mechanics and reality. How can it be explained?

Penrose (1989; 2004, cf. Bennett and Wiesner, 1992) suggested quantum entanglements are not mediated in a normal causal way, that non-local entanglement (quantum information, or “quanglement,” as Penrose terms it) should be thought of as able to propagate in either direction in time (into the past or into the future). Along similar lines, Aharonov and Vaidman (1990) also proposed that quantum state reductions send quantum information both forward and backward in what we perceive as time, “temporal non-locality.” However it is generally agreed that quantum information going backward in time cannot, by itself, communicate or signal ordinary classical information; it is “acausal.” This restriction is related to elimination of possible causal paradox (e.g., signaling backward in time to kill one's ancestor, paradoxically preventing one's birth). Indeed quantum information going forward in time is also considered acausal, unable to signal classical information either. In quantum cryptography and teleportation, acausal quantum information can only influence or correlate with classical information, but nonetheless greatly enhance capabilities of causal, classical processes.

**Figure 11:**
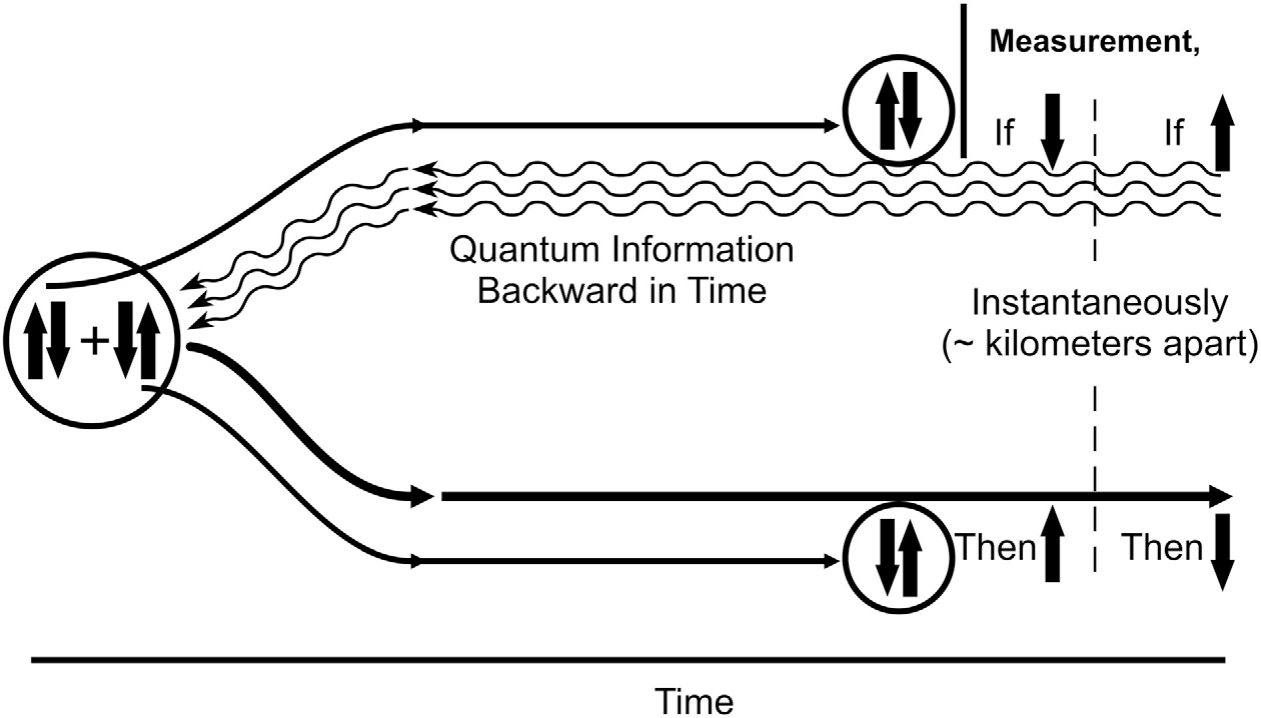
**Backward time in EPR entanglement.** The Einstein-Podolsky-Rosen (EPR) experiment verified by Aspect et al. (1982); Tittel et al. (1998), and many others. On the left is an isolated, entangled pair of superpositioned complementary quantum particles, e.g., two electrons in spin up and spin down states. The pair is separated and sent to two different, spatially-separated locations/measuring devices. The single electron at the top (in superposition of both spin up and spin down states) is measured, and reduces to a single classical state (e.g., spin down). Instantaneously its spatially-separated twin reduces to the complementary state of spin up (or vice versa). The effect is instantaneous over significant distance, hence appears to be transmitted faster than the speed of light. According to Penrose (2004; cf. Bennett and Wiesner, 1992), measurement/reduction of the electron at the top sends quantum information backward in time to the origin of the unified entanglement, then onward to the twin electron. No other reasonable explanation has been put forth.

Penrose suggested acausal backward time effects used in conjunction with classical channels could influence classical results in a way unattainable by classical, future-directed means alone, and that temporal non-locality and acausal backward time effects were essential features of entanglement. He suggested that in EPR (Figure 11), quantum information/quanglement from the measurement/state reduction moves backward in (what we “naively” perceive as classical) time to the unified pair, then to the complementary twin, influencing and correlating its state when measured. Can quantum backward referral happen in the brain?

## Orchestrated objective reduction (Orch OR)

**Figure 12:**
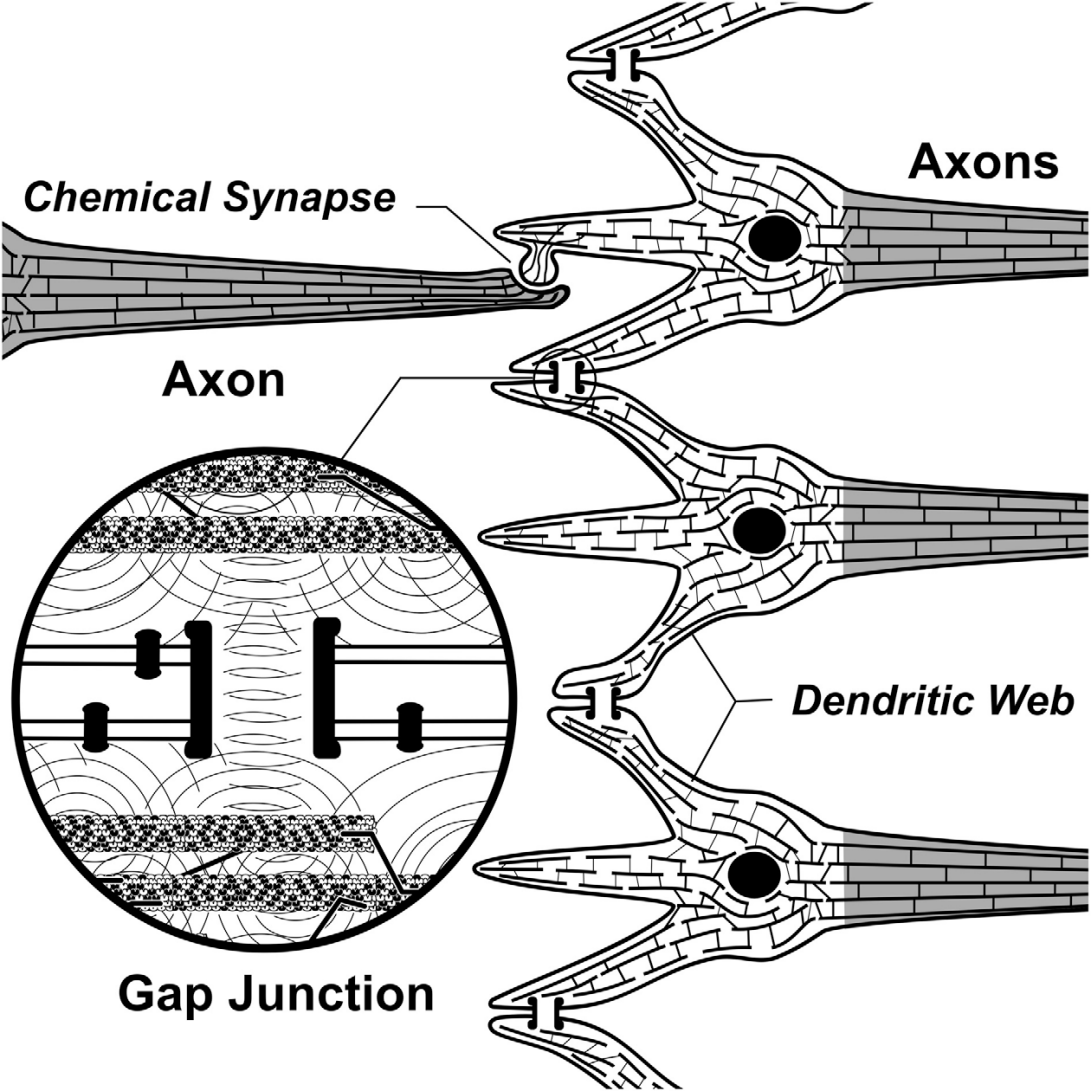
**Three toy neurons in an input/integration layer.** Adjacent dendrites are connected by gap junction electrical synapses in “dendritic web,” showing internal cytoskeletal microtubules connected by microtubule-associated proteins. Insert: communication/correlation between microtubules through gap junctions by electromagnetic or quantum entanglement, enabling collective integration among gap junction-connected, synchronized neurons and glia.

Penrose put forth OR as a mechanism for consciousness in physical science (the first, and still only specific proposal). For neurobiological implementation of OR, the Penrose–Hameroff model of “Orch OR” proposed quantum computations terminated by OR in microtubules within brain neurons, “orchestrated” by synaptic inputs, memory and other factors, hence “Orch OR” (Penrose and Hameroff, 1995, 2011; Hameroff and Penrose, 1996a,b; Hameroff, 1998, 2007). Starting with classical microtubule automata (e.g., Rasmussen et al., 1990) in which tubulins in microtubule lattices convey interactive bit states, e.g., of 1 or 0, and are thus capable of classical information processing (Figure 5B), Orch OR also proposed that quantum superpositioned tubulin bits, or “qubits,” e.g., of both 1 AND 0 compute via entanglement with tubulins in the same neuron, and also those in neighboring and distant neurons via gap junctions (Figure 12). The quantum computations evolve by the Schrödinger equation in entangled microtubules in dendrites and cell bodies during integration phases of gap junction-connected integrate-and-fire neurons. Entangled superpositions contribute to increasing gravitational self-energy E. When threshold is met by *E* = *ħ*/*t*, a conscious moment occurs as entangled tubulin qubits simultaneously undergo OR to classical tubulin states which then proceed to trigger (or not trigger) axonal firings, and adjust synapses. Microtubule quantum computations can thus be the “x-factor” in integration regulating axonal firing threshold. Compatible with known neurophysiology, Orch OR can account for conscious causal control of behavior.

Entangled superpositions leading to OR and moments of consciousness by *E* = *ħ*/*t* are seen as sequential, only one “consciousness” occurring in the brain at any one time (except perhaps for “split-brain” patients, or those with other cognitive disorders). Superpositions outside the largest, most rapidly evolving gap junction-connected web may decohere randomly, or continue and participate in a subsequent moment of consciousness. The results of each Orch OR conscious moment set initial conditions for the next.

By *E* = *ħ*/*t*, superposition of about 2 × 10^10^ tubulins would reach threshold at *t* = 25 ms, as in 40 Hz gamma synchrony, 40 conscious moments/s. Depending on the percentage of tubulins involved per neuron, this would entail thousands to hundreds of thousands of gap junction-connected neurons per conscious moment at 40 Hz as the NCC (Figure 12). With specific neuronal distributions and brain regions defined by gap junction openings and closings, synchronized “dendritic webs” as the NCC can move and redistribute moment to moment. Within the NCC, consciousness by *E* = *ħ*/*t* may occur on a spectrum of frequencies, at different fractal-like scales of brain activity (He et al., 2010), with deeper order, finer scale entangled processes in microtubules correlating with high frequency, high intensity experience, and larger proportions of brain involvement.

**Figure 13:**
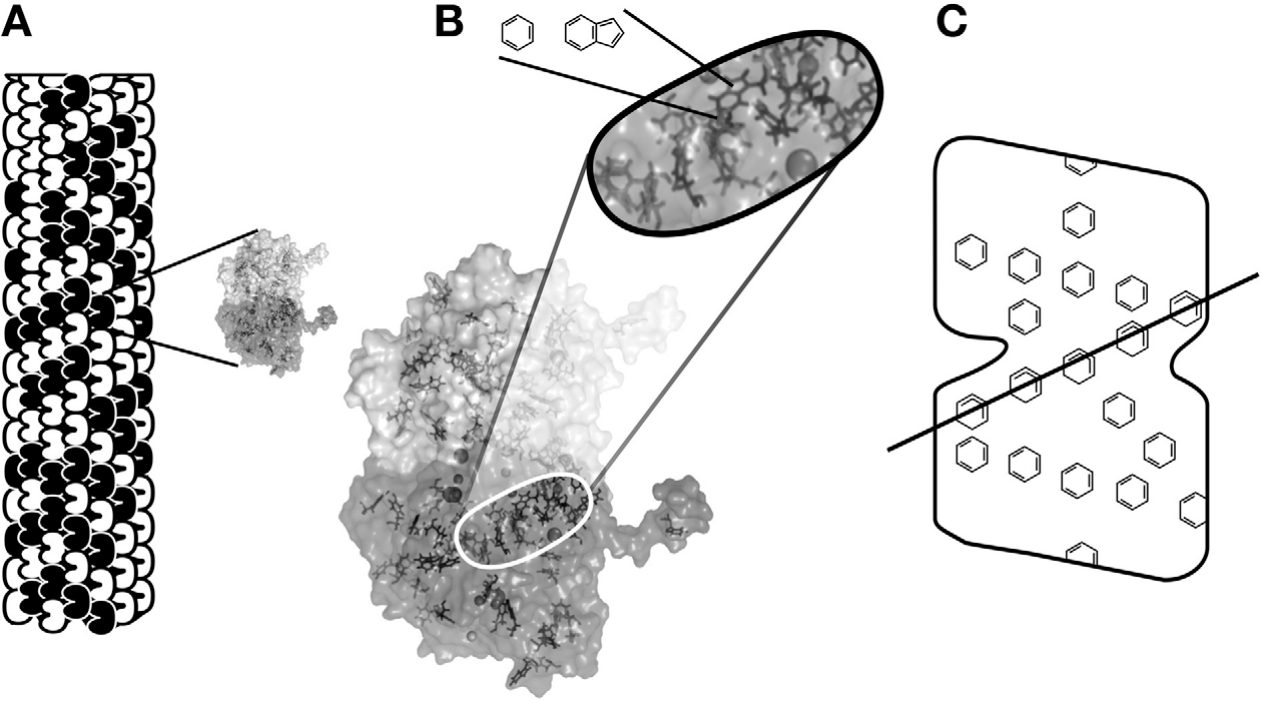
**(A)** A microtubule, a cylindrical lattice of peanut-shaped tubulin proteins, with molecular model of enlarged single tubulin with C-termini tails (Craddock et al., 2012c). **(B)** Tubulin dimer, lower C terminus tail visible. Interior blowup shows aromatic rings clustered in a linear groove, and further blowup of ring structures. **(C)** Approximate locations of resonance rings suggesting trans-tubulin alignments (see Figure 14A).

Proteins can act as quantum levers, able to amplify quantum effects into particular classical states (Conrad, 1994). Orch OR suggests that tubulin states and superpositions are initiated by electron cloud dipoles (van der Waals London forces) in clusters of aromatic resonance rings (e.g., in amino acids tryptophan, phenylalanine, tyrosine, Figures 13A–C). London force dipoles are inherently quantum mechanical, tending to superposition. They also mediate effects of general anesthetic gases which act in aromatic clusters (“hydrophobic pockets”) in neuronal proteins including tubulin to selectively erase consciousness (Hameroff, 2006). This suggests a deeper order, finer scale component of the NCC.

Electron movements of one nanometer, e.g., in a London force dipole oscillation, displace atomic nuclei by one Fermi length, 10^−15^ m, the diameter of a carbon atom nucleus (Sataric et al., 1998), and also the superposition separation distance required for gravitational self-energy E in Orch OR (Hameroff and Penrose, 1996a,b). Thus London forces can induce superposition of an entire protein/tubulin mass, albeit by an extremely tiny separation distance. Nonetheless the protein-level (rather than electron only) superposition separation engenders significant gravitational self-energy *E*, and thus by *E* = *ħ*/*t*, usefully brief durations of time *t* for conscious moments and actions.

Orch OR has been criticized on the basis of decoherence in the “warm, wet and noisy” brain, preventing superposition long enough to reach threshold (Tegmark, 2000; cf. Hagan et al., 2001). But subsequently plant proteins have been shown to routinely use electron superposition for chemical energy (Engel et al., 2007). Further research has demonstrated warm quantum effects in bird brain navigation (Gauger et al., 2011), ion channels (Bernroider and Roy, 2005), sense of smell (Turin, 1996), DNA (Rieper et al., 2011), protein folding (Luo and Lu, 2011), and biological water (Reiter et al., 2011). Microtubules (Sahu et al., 2012) appear to have kilohertz and megahertz resonance related to enhanced (?quantum) conductance through spiral pathways.

**Figure 14:**
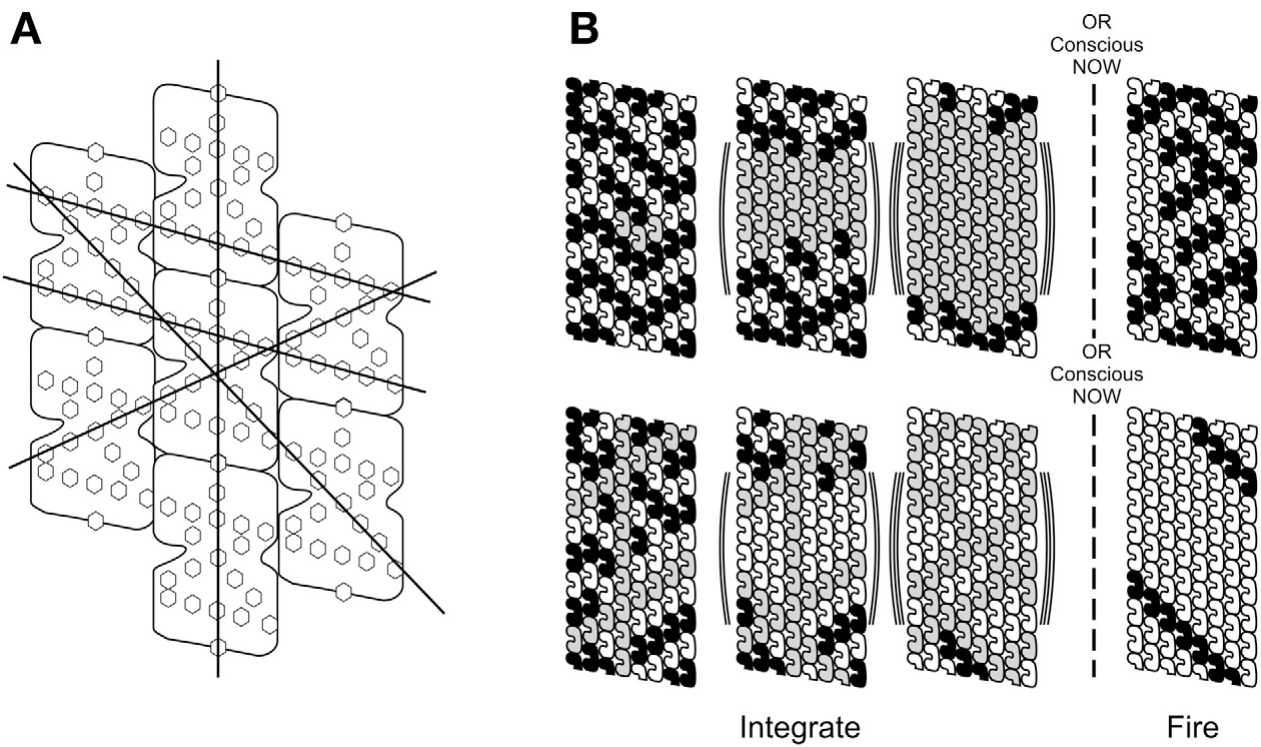
**(A)** Alignment of aromatic ring structures in tubulins and through microtubule lattice suggests different helical pathways, possible macroscopic “quantum highways” e.g., following the Fibonacci sequence in the A lattice. **(B)** Top: superpositioned tubulins (gray) increase through first three steps (neuronal integration) until threshold is met by *E* = *ħ*/*t*, resulting in Orch OR, a conscious moment, and selection of classical tubulin states which may trigger axonal firing. **(B)** Bottom: same as **(A)**, but with topological qubits, i.e., different helical pathways represent information. One particular pathway is selected in the Orch OR conscious moment.

Conductance pathways through aromatic ring arrays in each tubulin aligned with neighbor tubulin arrays following spiral geometry in microtubule lattices (Figure 14A) allow helical macroscopic “quantum highways” through microtubules (Figure 14A) suitable for topological quantum computing (Kitaev, 1997; Hameroff et al., 2002; Penrose and Hameroff, 2011). With particular spiral pathways as topological qubits (“braids”) rather than individual tubulins, overall microtubule information capacity is reduced, each topological bit/qubit pathway requiring many tubulins (Figure 14B, Bottom). But topological qubits are robust, resist decoherence, and reduce to classical helical pathways (or combinations) which can, with each conscious moment, regulate synapses and trigger axonal firings.

**Figure 15:**
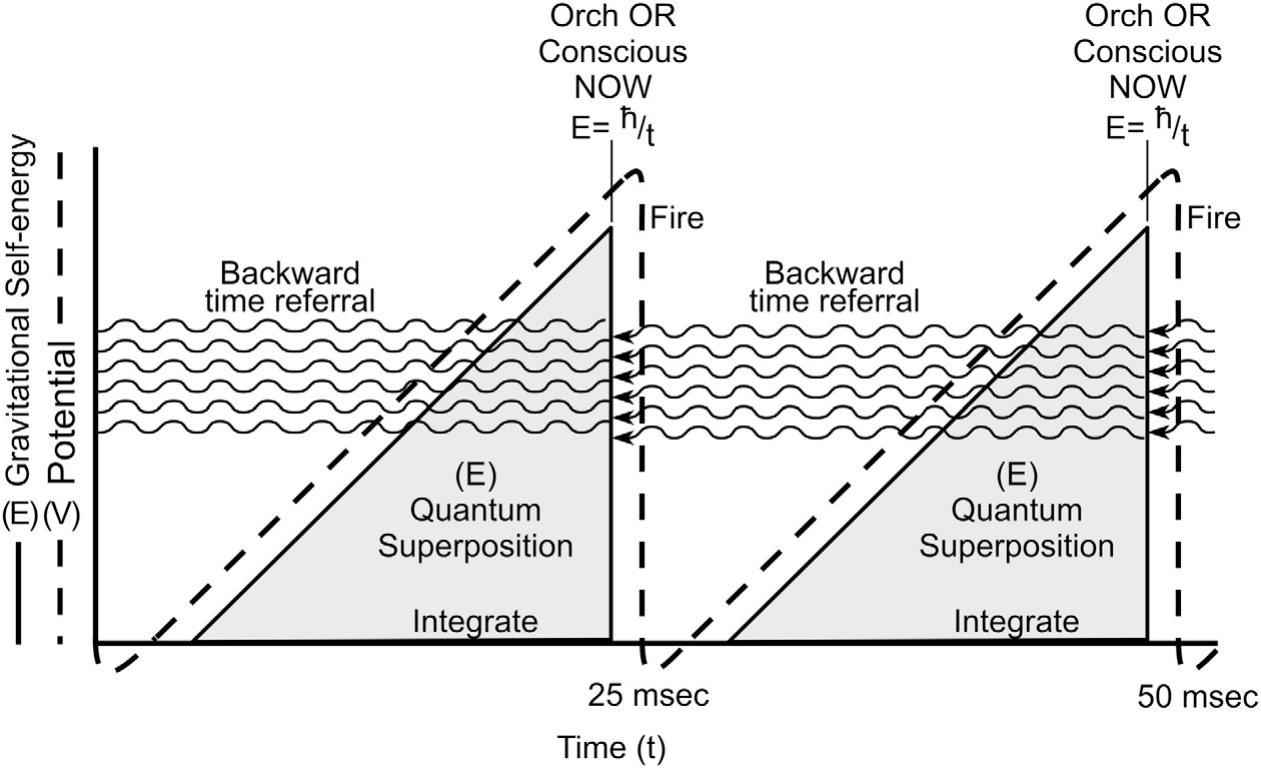
**Two Orch OR events (solid lines) underlie integrate-and-fire electrophysiology (dotted lines) in neurons.** Orch OR and conscious moments occur here at *t* = 25 ms (gamma synchrony), with *E* then equivalent to superposition of approximately 2 × 10^10^ tubulins. Each Orch OR moment occurs with conscious experience, and selects tubulin states which can then trigger axonal firings. Each Orch OR event can also send quantum information backward in perceived time.

In Figure 15, two Orch OR conscious moments underlie gamma synchrony electrophysiology in an integrate-and-fire neuron. Quantum superposition E evolves during integration, increasing with time until threshold is met at *E* = *ħ*/*t* (*t* = 25 ms), at which instant an Orch OR conscious moment occurs (intensity proportional to *E*), and classical states of tubulin are selected which can trigger (or not trigger) axonal firings which control actions and behavior (as well as regulate synaptic strength and record memory).

Each Orch OR quantum state reduction also causes temporal non-locality, sending quantum information/quanglement (with gravitational self-energy *E*) backward in what we perceive as classical time, integrating with forward-going *E* to help reach *E* = *ħ*/*t*, perhaps earlier than would otherwise occur (Figure 2B). As described previously, Orch OR temporal non-locality and backward time referral of quantum information can provide real-time conscious causal control of voluntary actions (Figure 6; cf. Wolf, 1998; Sarfatti, 2011).

Do backward time effects risk causal paradox? In classical physics, the cause of an effect must precede it. But backward-going quanglement is acausal, only able to influence or correlate with information in a classical channel, e.g., as occurs in quantum entanglement, cryptography and teleportation. And according to some quantum interpretations, backward time effects can't violate causality if they only alter past events whose subsequent effects had not been consciously observed (“If a tree falls ….”). In the experimental studies cited here (Libet, pre-sentiment/Bem, delayed choice) backward referral itself is non-conscious (though Libet refers to it as “subjective experience”) until reduction occurs in the present. There is no causal paradox.

If conscious experience is indeed rooted in Orch OR, with OR relating the classical to the quantum world, then temporal non-locality and referral of acausal quantum information backward in time is to be expected (Penrose and Hameroff, 2011). Temporal non-locality and backward time referral can rescue causal agency and conscious free will.

## Conclusion: how quantum brain biology can rescue conscious free will

Problems regarding conscious “free will” include: (1) the need for a neurobiological mechanism to account for consciousness and causal agency, (2) conscious perceptions apparently occurring too late for real-time conscious responses, and (3) determinism. Penrose–Hameroff “Orch OR” is a theory in which moments of conscious choice and experience are identified with quantum state reductions in microtubules inside neurons. Orch OR can help resolve the three problematic issues in the following ways.

### A mechanism for consciousness and causal agency

Orch OR is based on sequences of quantum computations in microtubules during integration phases in dendrites and cell bodies of integrate-and-fire brain neurons linked by gap junctions. Each Orch OR quantum computation terminates in a moment of conscious experience, and selects a particular set of tubulin states which then trigger (or do not trigger) axonal firings, the latter exerting causal behavior. Orch OR can in principle account for conscious causal agency.

### Does consciousness come too late?

Brain electrical activity appearing to correlate with conscious perception of a stimulus can occur *after* we respond to that stimulus, seemingly consciously. Accordingly, consciousness is deemed epiphenomenal and illusory (Dennett, 1991; Wegner, 2002). However evidence for backward time effects in the brain (Libet et al., 1983; Bem, 2012; Ma et al., 2012), and in quantum physics (e.g., to explain entanglement, Penrose, 1989, 2004; Aharonov and Vaidman, 1990; Bennett and Wiesner, 1992) suggest that quantum state reductions in Orch OR can send quantum information backward in (what we perceive as) time, on the order of hundreds of milliseconds. This enables consciousness to regulate axonal firings and behavioral actions in real-time, when conscious choice is felt to occur (and actually does occur), thus rescuing consciousness from necessarily being an epiphenomenal illusion.

### Determinism

Is the universe unfolding (in which case free will is possible), or does it exist as a “block universe” with pre-determined world-lines, our actions pre-determined by algorithmic processes? In Orch OR, consciousness unfolds the universe. The selection of states, according to Penrose, is influenced by a non-computable factor, a bias due to fine scale structure of spacetime geometry. According to Orch OR, conscious choices are not entirely algorithmic.

Orch OR is a testable quantum brain biological theory compatible with known neuroscience and physics, and able to account for conscious free will.

### Conflict of interest statement

The author declares that the research was conducted in the absence of any commercial or financial relationships that could be construed as a potential conflict of interest.
